# Supplementary material for: Multi-omic brain and behavioral correlates of cell-free fetal DNA methylation in macaque maternal obesity models
Source: Nat Commun. 2022 Sep 21;13:5538. doi: 10.1038/s41467-022-33162-7 (PMC9492781; doi:10.1038/s41467-022-33162-7)
Supplement: Supplementary file 1 — Supplementary Information [file 41467_2022_33162_MOESM1_ESM.pdf]

Multi-omic brain and behavioral correlates of cell-free fetal DNA methylation in macaque maternal obesity models

Supplementary Table 1: Group comparisons of **a)** background variables and **b)** body weights of dams during pregnancy (kg).

A. Background variables.

	N	Dam BCS (score)	Sire BCS[1] (score)	Dam age (years)	Delivery type (vaginal/c-section)	Fostered (yes/no)
Obese	7	3.6±.14	2.9±0.1	10.46±.44	5/2	5/2
Control	6	2.2±.17****	2.8±0.3	9.05±.64	4/2	3/3
Caloric restriction	5	3.5±.00	3.5±0.4	12.23±.93	5/0	5/0
Pravastatin	7	3.7±.15	2.8±0.2	12.16±.56	6/1	6/1

Mean±sem; BCS= Body Composition Score
****p<.001 ANOVA with Dunnett's post hoc analysis of all groups compared to Obese group.
[1]mean of 5 values obtained during the study; one sire in the caloric restriction group had only one BCS.

B. Body weights of dams during pregnancy (kg)

	N	GD45	GD90	GD120	GD150
Obese	7	10.82±.44	10.94±.36	11.18±.40	11.51±.46
Control	6[1]	7.65±.41*	8.33±.38**	8.49±.42***	8.89±.43****
Caloric restriction	5	9.69±0.83	10.00±.89	9.99±.88	10.14±.83
Pravastatin	7	9.79±.29	9.80±.35	9.98±.41	10.32±.47

Mean±sem; GD=gestation day
*p=.0006, **p=.0041, ***p=.0032, ****p=.0057 ANOVA with Dunnett's post hoc analysis of all groups compared to Obese group.
[1]N=5, GD45, GD90

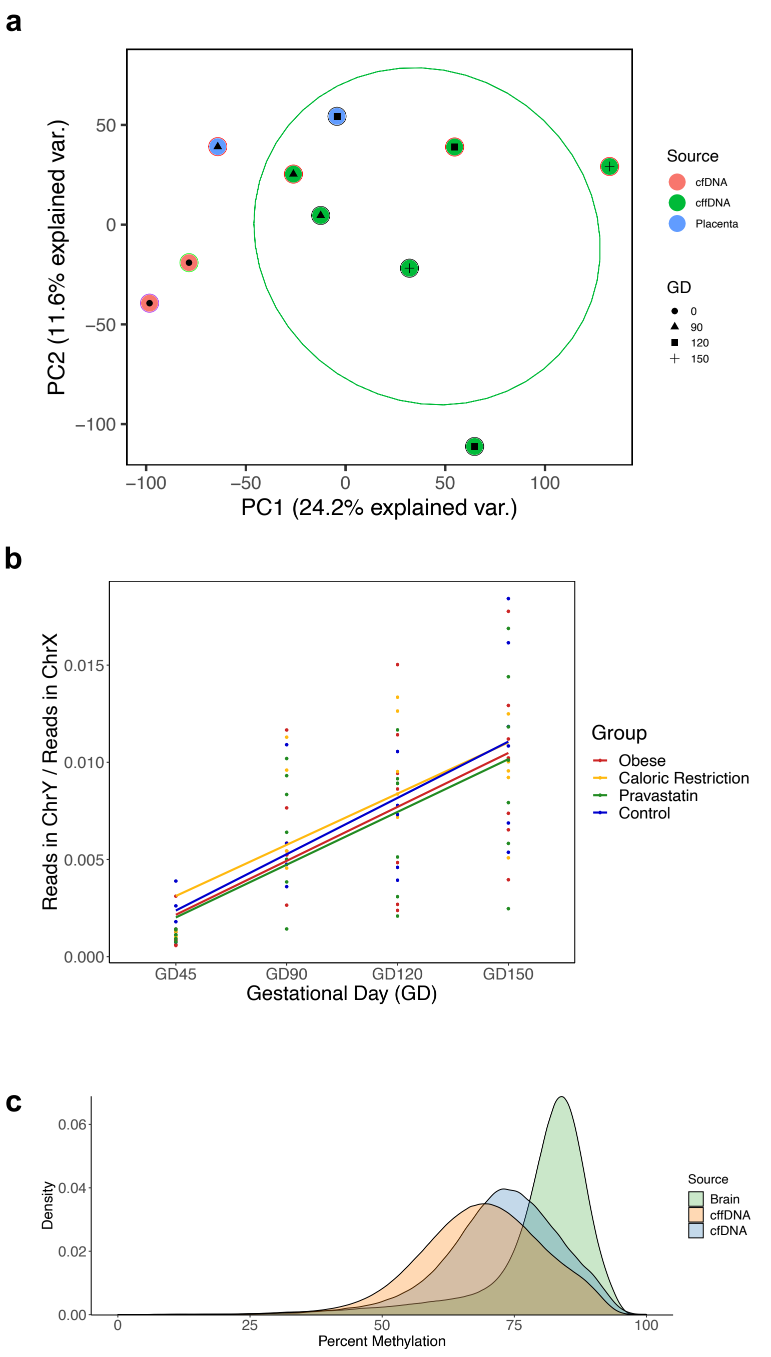

Supplementary Fig. 1: cffDNA technical confirmation experiments from the pilot and main experiments. **a)** Principal component analysis (PCA) of smoothed methylation levels from gene regulatory and gene body regions (+5 kb to -1 kb from the gene body) comparing cffDNA, cell-free DNA (cfDNA) from non-pregnant mothers, and placental biopsies from different pregnancy time points in a lower coverage pilot experiment. The outermost color around each dot represents the individual animal. **b)** Line plot of the ratio of Y chromosome reads over X chromosome reads for the cffDNA methylomes across all pregnancy timepoints for the different groups from the main experiment. **c)** Density plots of global DNA methylation levels (20 kb windows) in cffDNA, cfDNA, and brain from the main experiment.

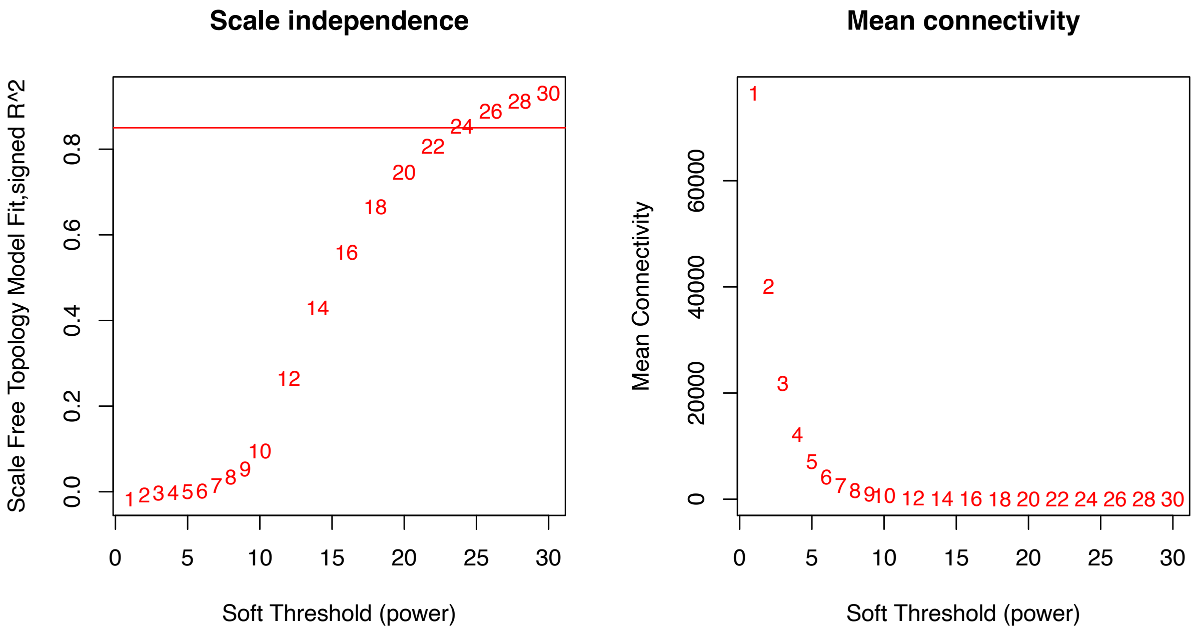

Supplementary Fig. 2: Analysis of scale-free topology used to determine the soft-thresholding power for infant hippocampal DNA methylation network construction.

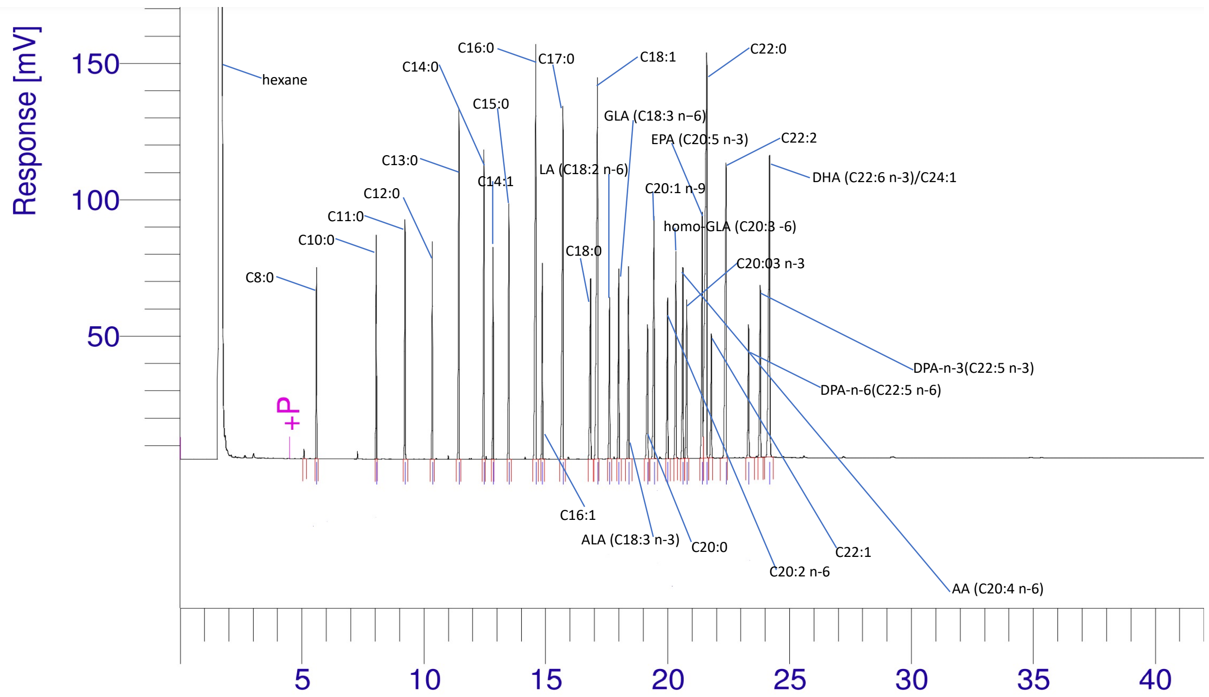

Supplementary Fig. 3: Chromatogram of 29 FAME standards used for lipidomic analyses.
