# Supplementary material for: Multi-omic brain and behavioral correlates of cell-free fetal DNA methylation in macaque maternal obesity models
Source: Nat Commun. 2022 Sep 21;13:5538. doi: 10.1038/s41467-022-33162-7 (PMC9492781; doi:10.1038/s41467-022-33162-7)
Supplement: Supplementary file 3 — Description of Additional Supplementary Files [file 41467_2022_33162_MOESM3_ESM.pdf]

Description of Additional Supplementary Files

Supplementary Data 1: Immunological and metabolomic measurements of maternal blood across pregnancy.

Supplementary Data 2: Infant lipidomic and metabolomic measurements.

Supplementary Data 3: Annotated regions from the infant hippocampus blue and yellow module maternal obesity networks.
